# The dual role of the CD95 and CD95L signaling pathway in glioblastoma

**DOI:** 10.3389/fimmu.2022.1029737

**Published:** 2022-11-24

**Authors:** Yanrui Zhang, Taian Jin, Zhangqi Dou, Boxing Wei, Buyi Zhang, Chongran Sun

**Affiliations:** ^1^ Department of Neurosurgery, Second Affiliated Hospital, School of Medicine, Zhejiang University, Hangzhou, China; ^2^ Department of Pathology, The Second Affiliated Hospital, School of Medicine, Zhejiang University, Hangzhou, China; ^3^ Key Laboratory of Precise Treatment and Clinical Translational Research of Neurological Diseases, Hangzhou, Zhejiang, China; ^4^ Clinical Research Center for Neurological Diseases of Zhejiang Province, Hangzhou, Zhejiang, China

**Keywords:** CD95, apoptosis, glioblastoma, TIL death, tumor immunotherapy

## Abstract

Binding of CD95, a cell surface death receptor, to its homologous ligand CD95L, transduces a cascade of downstream signals leading to apoptosis crucial for immune homeostasis and immune surveillance. Although CD95 and CD95L binding classically induces programmed cell death, most tumor cells show resistance to CD95L-induced apoptosis. In some cancers, such as glioblastoma, CD95-CD95L binding can exhibit paradoxical functions that promote tumor growth by inducing inflammation, regulating immune cell homeostasis, and/or promoting cell survival, proliferation, migration, and maintenance of the stemness of cancer cells. In this review, potential mechanisms such as the expression of apoptotic inhibitor proteins, decreased activity of downstream elements, production of nonapoptotic soluble CD95L, and non-apoptotic signals that replace apoptotic signals in cancer cells are summarized. CD95L is also expressed by other types of cells, such as endothelial cells, polymorphonuclear myeloid-derived suppressor cells, cancer-associated fibroblasts, and tumor-associated microglia, and macrophages, which are educated by the tumor microenvironment and can induce apoptosis of tumor-infiltrating lymphocytes, which recognize and kill cancer cells. The dual role of the CD95-CD95L system makes targeted therapy strategies against CD95 or CD95L in glioblastoma difficult and controversial. In this review, we also discuss the current status and perspective of clinical trials on glioblastoma based on the CD95-CD95L signaling pathway.

## Introduction

Glioblastoma (GBM) is the most common and aggressive malignancy in the central nervous system, the efficiency of standard treatment (surgical, radiotherapy, and temozolomide chemotherapy) is limited, and the prognosis is generally poor. Regulating cell death is an attractive target for cancer therapy. CD95 (FAS; APO-1; TNFRSF6) is a member of the tumor necrosis factor (TNF) receptor family of membrane surface proteins ubiquitously expressed in tissues, and its cognate ligand CD95L (FasL/CD178) is expressed primarily by cells of the immune system such as natural killer (NK) cells and activated T lymphocytes. CD95-mediated apoptosis helps to maintain immune system homeostasis and promotes the elimination of malignant cells (1). Although immune cells use CD95L as a mechanism for killing cancer cells, most tumor cells are resistant to CD95L-induced apoptosis. In some cancers, such as GBM, CD95-CD95L can exhibit an atypical function. Previous studies have suggested that CD95 is a specialized death receptor; however, the nonapoptotic functions of CD95 signaling have recently been found to promote tumor cell growth and migration (2). It is essential to explore the dual function of CD95 and the corresponding mechanisms for its application in cancer therapy.

## CD95-mediated apoptosis signaling pathway

**Figure 1:**
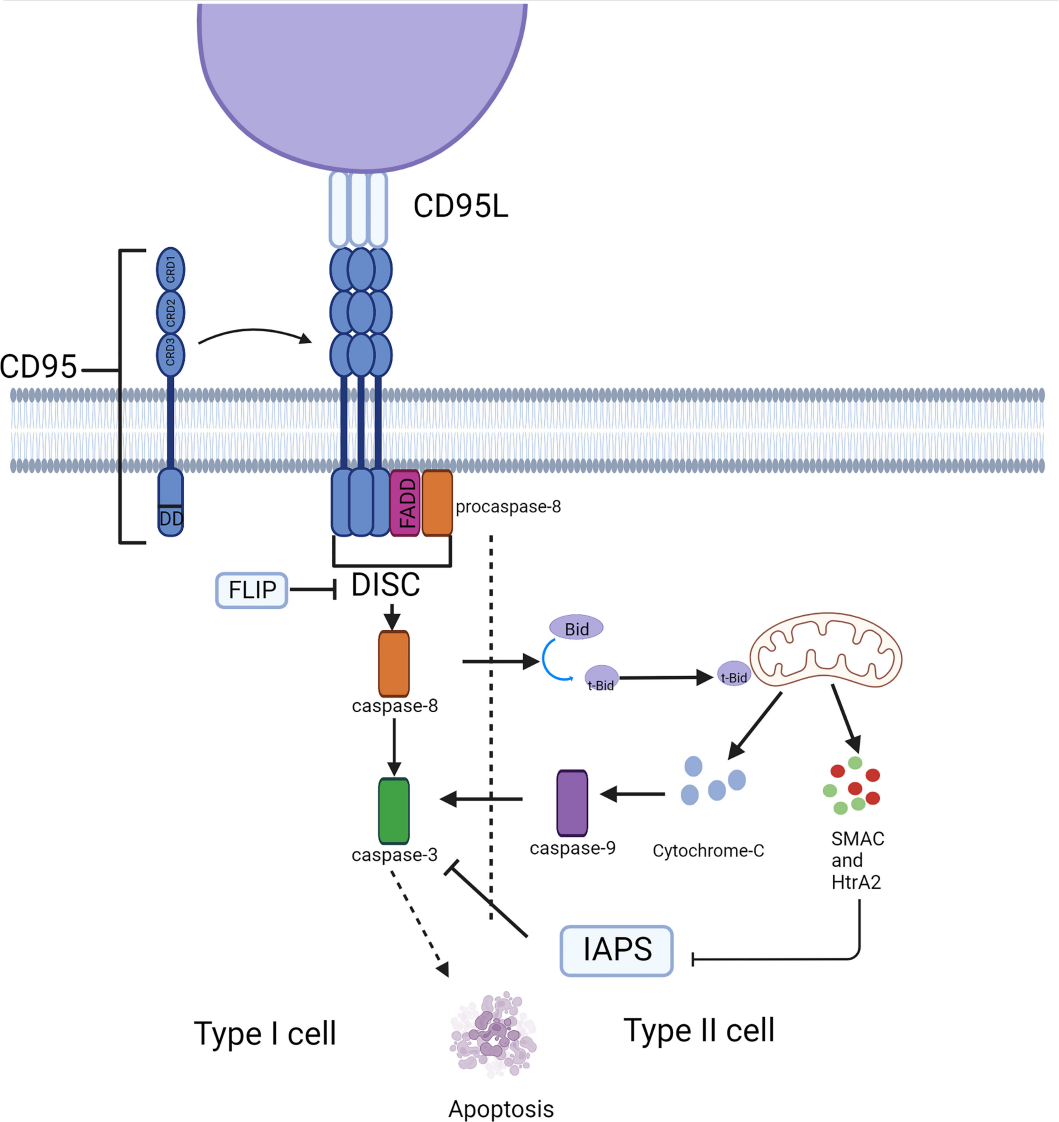
Typical apoptosis signaling pathway mediated by CD95. CD95 induces cell apoptosis through the caspase cascade in different cell types. This process depends on the involvement of mitochondria in type II cells.

CD95, a type 1 transmembrane protein, belongs to the TNF receptor superfamily. The extracellular region of CD95 contains three cysteine-rich domains (CRD) that define the ability to recognize and bind CD95 and CD95L. CRD2 and CRD3 of CD95 are involved in CD95L binding, and the preligand assembly domain (PLAD) overlapping CRD1 which is near the N-terminus is required for the assembly of CD95 trimer (3). The death domain (DD) in the intracellular segment of the CD95 molecule is a conserved region of about 80 amino acids, which is crucial for the transmission of apoptotic signals (4). Its ligand, CD95L, is a type II transmembrane protein that can be trimerized by the C-terminal TNF homology domain (THD). The apoptotic ability would be activated when CD95L binds to the death receptor CD95, the binding drives the aggregation of CD95 trimers, which causes DD to cluster together and attracts another protein in the cytosol with the same DD, called Fas-associated protein with death domain (FADD) (5). FADD then ligates the inactive zymogen form of caspase-8 (procaspase-8) through an N-terminal death effector domain (DED) (6), together they constitute the death-inducing signaling complex (DISC) (7), which recruits additional procaspase-8 molecules. Multiple procaspase-8 molecules bind through their tandem DED, thus forming the assembly of DED/caspase-8 filaments (8, 9). The formation of the DED chain drives the dimerization and self-cleavage of procaspase-8 that converts procaspase-8 into activated heterotetramer caspase-8, thus launching executioner caspases (e.g., caspase-3,-6, and-7), ultimately triggering a signaling cascade of apoptosis (**Figure 1**). However, activation of DR does not produce sufficient amounts of activated caspase-8 to trigger apoptosis in some cases, as has been observed in hepatocytes, pancreatic beta cells, and most cancer cells. In these so-called ‘Type II cells’, mitochondria-dependent intrinsic apoptotic pathways are required to amplify DR-mediated apoptotic signaling. This depends on whether XIAP expression is sufficient to block the enzymatic activities of caspase-3,-9, and -7 (10). The BH3 interacting domain death agonist (Bid) is cleaved by caspase-8 to generate a truncated form (tBid). Translocation of tBid to mitochondria, where it activates pro-apoptotic members of the Bcl-2 family Bax and Bak (11), can mediate mitochondrial outer membrane permeabilization (MOMP), thus releasing cytochrome C, Smac/Diablo and Htra2/OMI into the cytosol (12). This process can also be performed by tBid itself, without relying on Bax/Bak activation (12). The apoptosome is made up of cytochrome C, apoptosis-promoting factor-1 (Apaf1), and procaspase-9 (13), Smac, and Htra2 remove the inhibitory effect of XIAP on caspase-3 (14, 15), leading to caspase-3 activation induction of apoptosis. Thus, unlike type I cells, type II cells can be rescued from CD95-induced apoptosis by inhibiting MOMP (16, 17), for example, GBM overexpress members of the anti-apoptotic Bcl-2 family (18, 19) and downregulate the expression of BAX (20) and pro-apoptotic Bcl-2 proteins. Similarly, disruption of CD95 pro-apoptotic signaling cascades that evade apoptosis has been observed in GBM.

## CD95-mediated nonapoptotic signaling

### The anti-apoptotic function of CD95

**Figure 2:**
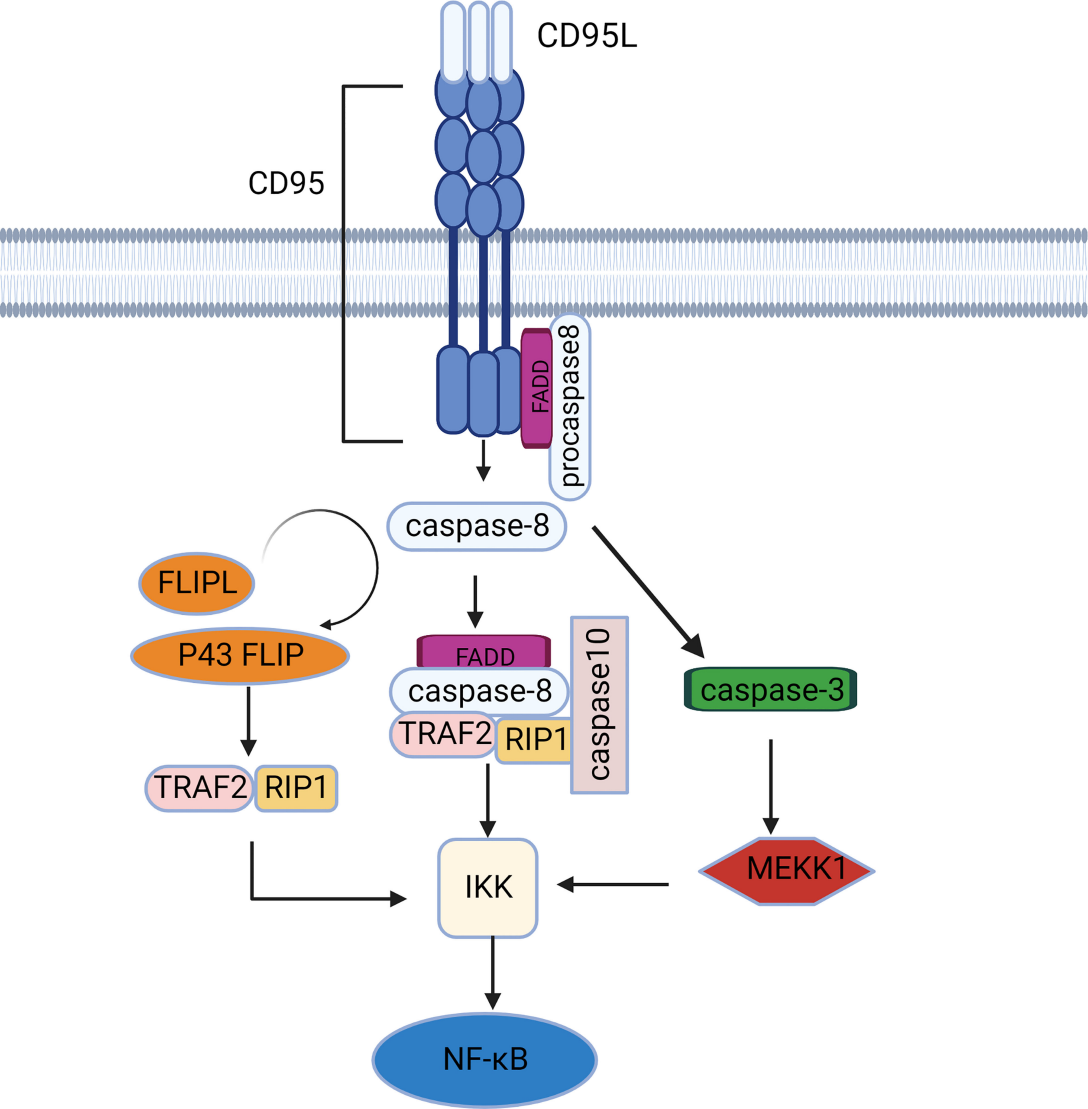
The NF-κB signaling pathway mediated by CD95. CD95 induces caspase-dependent activation of the NF-κB pathway *via* IKK activation. There are 2 major IKK activation pathways: the RIP1/TRAF2-dependent pathway and the MEKK1 activation pathway.

**Figure 3:**
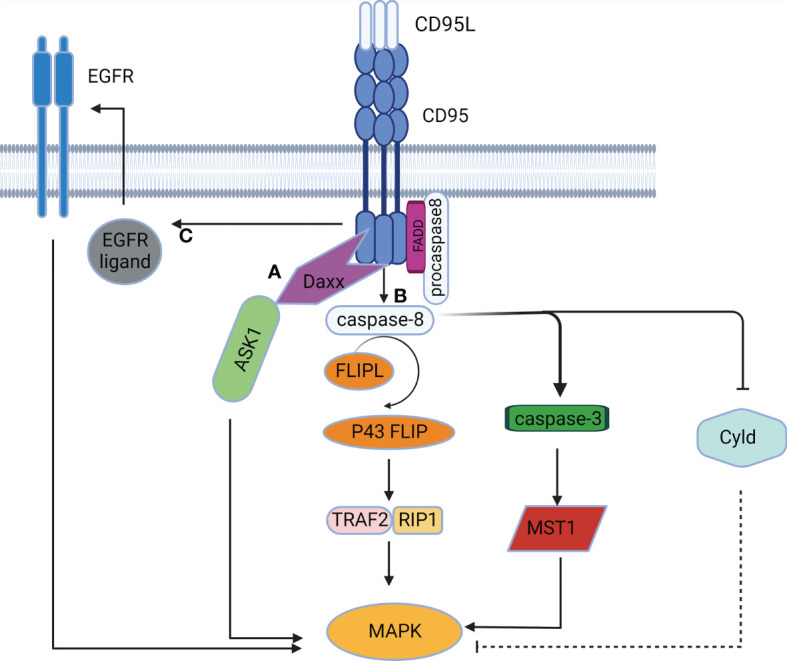
The MAPK signaling pathway mediated by CD95. **(A)** CD95 induces DD-mediated caspase-independent activation of the MAPK pathway. **(B)** CD95 induces caspase-dependent activation of the MAPK pathway. **(C)** CD95 induces activation of the MAPK pathway in a DD-independent manner by stimulating EGFR.

**Figure 4:**
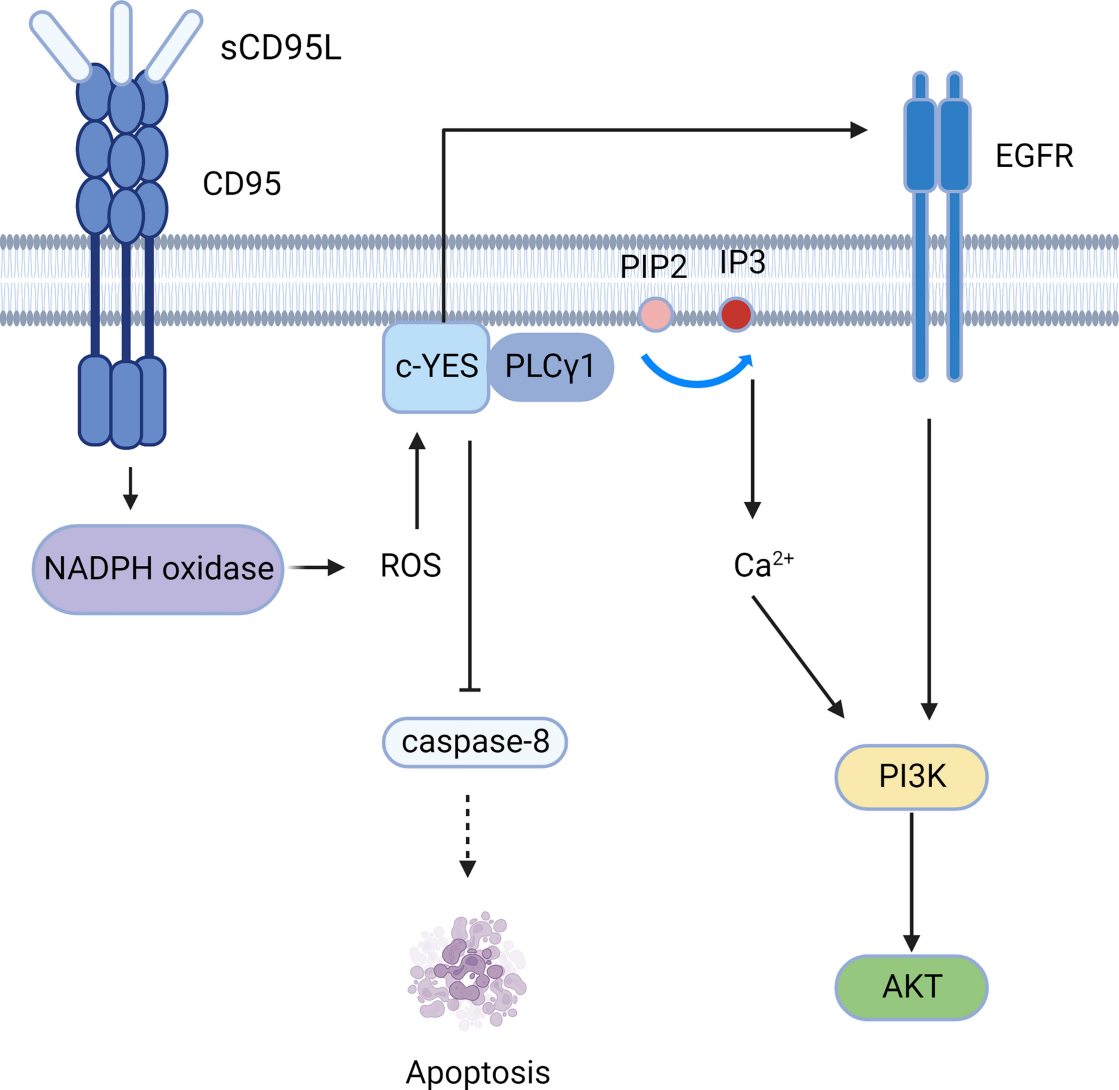
The PI3K-AKT signaling pathway mediated by CD95. CD95 induces the c-YES/calcium/PI3K pathway and c-YES/EGFR/PI3K pathway to mediate cell migration.

The understanding of CD95 activity initially focused on its ability to induce apoptosis, but now it has switched to the nonapoptotic signaling pathway (**Figures 2**–**4**). Particularly in cancer, nonapoptotic CD95 signaling has been widely documented and has been associated with cancer cell growth, invasiveness, as well as cancer cell stemness (21).

Tumor volume and incidence of the mouse with ovarian and liver cancer decreased when CD95 expression was knocked down (22). CD95-mediated Sck/Shc2 activation is essential for pancreatic ductal adenocarcinoma growth and metastasis (23). Balta et al. found that for single tumor cells, CD95 activation promoted their apoptosis. Conversely, the activation of CD95 in tumor cells in tissue promotes their survival, possibly because cell-to-cell contact increases tyrosine kinase activity (24). Recent studies have also provided evidence for the presence of CD95L-independent but CD95-dependent signaling pathways in human glioma initiating cells (Gics) that maintain tumor malignancy (25). The lack of CD95 has been found to enhance immune regulation of tumor cells in triple-negative breast cancer mice, and this suppression of tumor growth is due to enhanced recruitment and activation of NK cells (26). The molecular mechanisms have been elucidated. Independent of CD95L, the C-terminal region of CD95 binds to the Kip1 ubiquitination-promoting complex 2 (KPC2), which in turn recruits the ubiquitin ligase KPC1 and p65, a member of the NF-κB family. NF-κB1 (p105) is ubiquitinated by KPC1 and is degraded to p50 without the transactivation domain, forming a homodimer p50/p50 and thus, inhibits the NF-κB pathway. When CD95 is absent, p50 production is reduced and p65 is released from the plasma membrane to bind to p50 to form a heterodimer that activates the NF-κB pro-inflammatory signaling pathway and regulates cellular immunity (27).

### NF-κB signaling pathway

At rest, the inhibitor of NF-κB protein (IκB) combined with NF-κB in an inactive state, activation of IκB kinase (IKK) ubiquitinates, phosphorylates and eventually degrades IκB, transferring NF-κB from the cytoplasm to the nucleus (28). IKK activation is more dependent on caspase-8 activity than CD95 activity, although caspase-8 can be a downstream target of the CD95 signaling pathway (**Figure 2**). Caspase 8 recruits FADD, TRAF2, RIPK1, and E3 ubiquitin ligases into a complex called FADDosome (29), where effector proteins (e.g., RIP1, TRAF2) bind to the ubiquitin binding domain of IKK in a K63 ubiquitin chain-dependent manner, leading to NF-κB activation and subsequent secretion of pro-inflammatory cytokines. Caspase-10, a homolog of caspase-8, can also be recruited by FADD (30, 31). Early data suggest that caspase-10 is equivalent to caspase-8 as an initiator caspase, leading to cell apoptosis (32). However, recent studies have found that caspase-10 can restrain the activity of caspase-8 in DISC and transform apoptotic signaling into an NF-κB signaling pathway (33). This may be because caspase-10 is a necessary component for the assembly of the FADDosome (34). Furthermore, caspase-8 cleaves its homologue c-FLIPL to produce P43-FLIP that binds NF-κB signaling molecules such as RIP1, TRAF2, and TRAF60 in the absence of CD95 (35). Data also suggest that other cFLIP variants (full-length cFLIPL and cFLIPS) can activate IKK through the interaction of ubiquitinated proteins with IKKγ/NEMO (36). E3 ligases play a key role the degradation of IκB, processing of NF-κB precursors, and activation of the IKK complex. Therefore, the presence of deubiquitinating enzymes such as CYLD cleaves polyubiquitin chains, effectively inhibiting the NF-κB pathway (37, 38).

In addition to promoting inflammation and survival, CD95 controls glioma cell invasion by regulating matrix metalloproteinases (MMP)-2 activation through the NF-κB-TIMP-2 pathway (39). NF-κB is also involved in the expression of decoy receptor (DcR) 3, which inhibits the conduction of the CD95 signal (40). Tumor necrosis factor-related apoptosis-inducing ligand (TRAIL) is another DR ligand that is highly homologous to CD95L and also activates downstream pathways through FADD and caspase-8. We hypothesize that CD95L can also induce delayed activation of JNK and IKK through caspase-mediated activation of MEKK1 similar to TRAIL, independent of RIP1 and TRAF2 expression. Thus, activation or inhibition of the NF-κB pathway depends on the level of c-FLIPL. When cFLIP was expressed at a low level, fully activated caspase-8 cleaved RIP1 and caspase-3, which was sufficient to inhibit NF-κB signaling and induce apoptosis in type 1 cells. However, in type II cells where caspase-3 is not capable of inducing apoptosis, caspase-3 activates the MEKK1/IKK/NF-κB pathway. In contrast, when cFLIP is overexpressed, caspase-8 with limited activity cannot activate caspase-3, and at the same time, it will cleave RIP1 in a small amount to slow down NF-κB signaling conduction (41).

### MAPK signaling pathway

The MAPK chain transfers upstream signals to downstream responding molecules by sequential phosphorylation. The MAPK pathway acts as a pivot in cell proliferation, differentiation, apoptosis, and angiogenesis, and also participates in tumorigenesis (42). CD95 was initially found to activate the MAPK singling pathway in a caspase-independent manner. Death domain-associated protein (Daxx) served as the activator of the Jun NH2-terminal kinase (JNK) pathway. Activation of CD95 induced Daxx to interact with the MAP3K signaling kinase-1 ASK1, thus alleviating the inhibitory intramolecular interaction of ASK1 and activating its kinase activity (43). CD95 can also activate the MAPK singling pathway in a caspase-dependent manner. Caspase-8 and cFLIPL-procaspase-8 heterodimers can trigger MAPK by the formation of p43 FLIP (35), which is associated with the Raf-MAPK cascade reaction (44). Furthermore, caspase-8 can cleave Cyld (45), resulting in the activation of JNK, p38 and ERK signaling (46, 47). Caspase-3 and caspase-7 also participate in CD95-induced MAPK activation by cleaving the mammalian 20-like sterile serine/threonine kinase 1 (MST1) to generate active fragments of MAPK (48). Additionally, CD95 can activate the MAPK pathway independently of DD (**Figure 3**), since CD95 can induce EGFR activation to mediate subsequent activation of ERK (49). Furthermore, as expressed in the NF-κB signaling pathway above, CD95L can activate the JNK pathway in a TRAIL-like mechanism (41).

### PI3K-Akt signaling pathway

The soluble form of CD95L (sCD95L) binds to the receptor and cannot form a DISC, but instead forms a complex called motion-induced signaling complex (MISC); MISC produces ROS through NADPH oxidase (for example, Nox3) (50). Src kinases such as c-YES are subsequently activated, followed by recruitment of PLCγ1 to the plasma membrane, where PLCγ1 hydrolyzes PIP2 to produce IP3 and DAG. Then IP3 activates the intracellular calcium response. Calcium influx can be promoted by CD95-activated Orai1 channels, and elevated calcium concentrations activate DISC inhibitors, such as PKCβ2 (51). Therefore, CD95 can mediate tumor invasion and migration through the c-YES/calcium/PI3K pathway (52).Moreover, c-YES stimulates EGFR in the absence of EGF (53), resulting in activation of the PI3K/Akt signaling pathway (**Figure 4**). Apart from PLCγ1, Src kinases can phosphorylate other MISC components, including TRIP6, which is involved in NF-κB activation and cell migration (54). Src kinases also phosphorylate caspase-8 at tyrosine 380 (Y380), preventing downstream activation of the caspase apoptotic signaling cascade (55).

Tumor migration and invasion can also be mediated by membrane-bound CD95L (mCD95L). Aggregation of CD95, c-YES, and PI3K has been confirmed in GBM cells, and thus, activation of the PI3K/Akt/GSK3β/MMP pathways can mediate invasion (56).

## Disruption of CD95-mediated apoptotic signaling in GBMs

### Reduced membrane expression of the CD95 receptor

Simultaneous expression of CD95 and CD95L has been detected in GBMs (57), but GBM has often showed resistance to CD95-mediated apoptosis. Mutations in the CD95 gene have been detected in hematological tumors and some solid tumors, and most are clustered to exons 8 and 9, which encode a major part of the intracellular region of CD95, resulting in the impediment of FADD recruitment (58).

GBM can also modulate CD95 availability on the cell membrane to inhibit CD95-induced apoptosis. The protein tyrosine phosphatase non-receptor 13 (PTPN13), also known as FAP-1, is a widely studied inhibitor of CD95 (59). FAP-1 is highly expressed in GBM (60) and binds to CD95 and to endosome-associated trafficking regulator 1 (ENTR1), thus achieving the allowing the association of CD95 and ENTR1. ENTR1, in turn, promotes CD95 transport to lysosomes (61), sequestering CD95 from the cell membrane and therefore inhibiting cell apoptosis. Additionally, FAP-1 also mediates multiple CD95-independent signaling pathways to inhibit cell proliferation and migration in solid tumors, and thus, plays an important role in cell apoptosis (62).

Lipid rafts are dynamic microdomains of plasma membranes, rich in cholesterol and sphingomyelin (63). Lipid rafts act as a skeleton to isolate and localize cell signaling. After activation, CD95 is recruited into lipid rafts to promote protein-protein interactions and the transmission of apoptotic signals (64). CD95-mediated non-apoptotic PI3K/Akt signaling inhibits CD95 aggregation in lipid rafts (65), thereby promoting tumor survival. In addition, lipid rafts are responsible for the invasion of GBM by collecting CD44 (66) and connexin 43 (67).

The function of a protein can be affected by its post-translational modifications, such as glutathionylation, glycosylation, and nitrosylation, which are conducive to assembly of DISC; instead, phosphorylation inhibits the formation of DISC (68). Palmitoylation positions CD95 in rafts, which is required for effective internalization of the CD95 receptor and subsequent caspase cascade activation (69). The DHHC family is a group of proteins related to protein palmitoylation, and most of these proteins have protein acyltransferase activity. Most DHHC members are expressed at a higher level in GBM, and generally promote tumor survival by regulating CD95-independent signaling pathways (70, 71). In contrast, ZDHHC7, ZDHHC11, and ZDHHC22 are down-regulated in GBM cells (71). ZDHHC7 is the main palmitoyltransferase of residue Cys199 of CD95. Limited CD95 palmitoylation reduces its membrane localization, hindering its distribution in lipid rafts (72).

### Interference with CD95-CD95L binding

The phenomenon of gene alternative splicing refers to the formation of different transcripts due to various splicing methods of mRNA precursors (choosing different splicing site combinations). It is a basic and important regulatory mechanism in eukaryotes and controls the expression of cancer-related proteins (73). CD95 premRNA performs alternative splicing by cutting exon 6 which encodes the transmembrane domain to produce soluble isomers sCD95 (74). Soluble CD95 is antiapoptotic by competing with mCD95 for CD95L. Overexpression of sCD95 has been detected in a wide range of cancers (75, 76). Recently, hypoxic microenvironments were found to modulate CD95 splicing and promote the production of anti-apoptotic sCD95 mRNA (77), possibly due to reduced interactions of the splicing factor U2AF-RNA in hypoxic cells (78).

Similarly, CD95L is also available in two forms, but sCD95L is formed by MMP-mediated proteolytic cleavage. sCD95L cannot activate CD95 as mCD95L does (79). It cannot form oligomers with CD95 and, therefore, it cannot induce DISC formation (80). Inhibition of mCD95 cleavage by MMP enhances CD95-mediated apoptosis (81). In addition, it can activate motor signals, which will be described below.

DcR belongs to the TNF receptor family, it is a soluble protein associated with the suppression of death receptors, which overexpression leads to immune escape. DcR3 has been detected in malignant gliomas, and its abnormal expression can inhibit CD95L-induced apoptosis (82, 83). DCR3 relies on four CRDs at the N-terminal to bind CD95L, therefore, three DcR3 and CD95L trimer form the heterohexameric complex. By recognizing the invariant backbone and side chain atom of the ligand, DcR3 specifically binds to CD95L, LIGHT, and TL1A (84). Due to the lack of transmembrane domain, DcR3 disturbs the binding of these receptors to ligands through competitive inhibition, while it also conducts the signaling pathways for tumor cell growth, invasion and epithelial-mesenchymal transition (EMT) (85, 86). Furthermore, through its CD95-independent properties, DcR3 induces local immune suppression, mainly through suppression of Th1 and macrophage propensity to M2 in the tumor microenvironment (87). It was also found to promote effector T cell apoptosis by reverse signaling (88); thus, further enhancing the immune escape of tumor cells.

### Blocking caspase-8 activation

The recruitment of procaspase-8 by FADD and subsequent assembly of DISC determine the fate of cell apoptosis. Caspase-8 and FADD mRNA and protein expression levels were lower in GBM tissues than in normal brain tissues (89). Furthermore, caspase-8 was silenced by DNA methylation and gene deletion in pediatric neuroblastoma amplified with MYCN (90). Caspase-8 ability is also highly regulated by post-translational modifications (91), of which phosphorylation is typical (55).

The antiapoptotic protein c-FLIP is a protein containing a DED, which is a homologue of caspase-8 and is overexpressed in a variety of tumors (92–96). The three common isoforms are the short, Raji, and long isoforms of cFLIP (cFLIPS, cFLIPR, and cFLIPL). All of these proteins contain two death effector domains and use them to combine with FADD, recruiting caspase-8/-10 at the DISC. cFLIPL also has a caspase-like domain but lacks a key active site residue (97). CFLIPS has been verified as a dedicated inhibitor of apoptosis, which can form an inactive heterodimer with procaspase-8 (98), and the cleavage of procaspase-8 can be completely blocked (99). There is also evidence that cFLIPS can block caspase-8 activation by breaking the caspase-8 filament to prevent dimerization of the caspase-8 catalytic domain on the DISC (100).

The pro-apoptotic functions of cFLIPL were also verified. Pro-apoptotic activity is induced when expressed at low levels, and the formation of heterodimers with procaspase-8 allows the activation of caspase-8. It is worth noting that the formation of the procaspase-8: cFLIPL heterodimer is preferential to the procaspase-8 homodimer. However, when cFLIPL is highly expressed, c-FLIP without catalytic activity will compete with procaspase-8 for DISC, thus inhibiting apoptosis (101). In addition, a fragment named p43-FLIP is obtained from cleaved c-FLIPL. It interacts with RIP1 and TRAF2 to activate NF-κB (35). Conversely, the expression of c-FLIPL determines the direction of NF-κB as discussed above. It also plays a pseudo-enzyme function independent of caspase-8 (102). Thus, c-FLIPL balances cell survival and death in a complex manner.

For these reasons, the activity of caspase-8 is limited in tumors. Incomplete activation of caspase-8 in tumors contributes to the induction of minority MOMP (103), a limited number of mitochondria undergo MOMP, and the amount of cytochrome c/SMAC released is insufficient to trigger apoptosis, but sufficient to activate downstream caspases at sublethal levels. Subsequent deficient endonuclease activation causes the accumulation of DNA damage without cell death, destabilizing the genome, and promoting tumorigenesis (104, 105).

### Upregulation of apoptosis inhibitors

Inhibitors of apoptosis proteins (IAP), characterized by at least one baculoviral IAP repeat (BIR) domain, negatively regulate apoptosis by inhibiting caspase activation. It is frequently observed to be upregulated in GBM samples (106). There are eight proteins in the family, of which XIAP has the most potent antiapoptotic properties and can directly contact and inhibit caspase-3, 7, and 9. XIAP inhibitors have been shown to cause caspase-dependent apoptosis (107). Patients with GBM have a lower survival rate with higher expression of the expression of the XIAP protein (106).

Unlike XIAP, cIAP-1 and cIAP-2 could not directly inhibit caspases, cIAP-1, cIAP-2, as well as XIAP have the same RING domains and possess the Ubiquitin protein ligase (E3) Ubiquitin associated activity (UBA) domain, which mediates caspase-3 and caspase-7 (108). They cause SMAC ubiquitination and subsequent degradation (109). Survivin/BIRC5, the smallest member of the IAP family of proteins, contains only one BIR domain and is the most upregulated IAP in a variety of tumors (110). Survivin binds to Smac to prevent this molecule from inhibiting XIAP, but also exerts antiapoptotic activity based on the ability to protect XIAP from ubiquitination (111). Based on the characteristic inhibition of caspase activity by IAP, an IAP antagonist, also known as a Smac mimetic, has the potential to sensitize tumor cells to apoptosis. However, IAP also influences other cellular processes, such as the regulation of ubiquitin-dependent NF-κB activation for cell survival (112). The interaction with the tumor microenvironment and the crosstalk with various cell signaling pathways lead to the complex role of IAP in cancer and immune regulation (113). The therapeutic results of IAP antagonists need to be further studied.

### Powerlessness of endonucleases

Even if the executioner caspases are successfully activated, apoptosis may not be achieved. Apoptotic hydrolysis of DNA requires endonuclease DFF40/CAD, and activation of DFF40/CAD requires its inhibitor ICD to be cleaved and inhibited by caspase-3. This step is limited in the cytosol, and thus the cytosolic level of DFF40/CAD is a determinant for achieving a complete apoptotic reaction (114). Due to abnormal accumulation of DFF40/CAD protein in the nucleus, GBM cells may not be able to undergo apoptosis (115).

## Immune privilege and tumor counterattack

CD95 is ubiquitously expressed in the human body and is presented as low tissue specificity. In contrast, CD95L is characteristically expressed in immune cells such as cytotoxic T lymphocytes and NK cells and has long been recognized an essential part of immune homeostasis and immune elimination. CD95L has also been reported to be constitutively expressed in immune-privileged tissues such as the eyes and testes. They mediate apoptosis of immune cells through CD95L, which renders them unable to respond to foreign antigens, including graft antigens (116). Astrocytes in healthy human brains do not express CD95L, but it is expressed by astrocytoma (117). Therefore, it has been proposed that tumors can desensitize themselves to apoptosis and express CD95L to mediate immune escape and realize immune counterattack. This effect is called a ‘tumor counterattack’. However, contradictory phenomena such as rejection and immune induction caused by CD95L expression in grafts or tumor cells have also been reported (118). In recent years, there has been a more profound and comprehensive understanding of tumor counterattack, whereby immune cell killing is not mediated by tumor CD95L, but by CD95L expressed in the tumor microenvironment. CD95L has been reported to be expressed in the human tumor endothelium. These CD95L-expressing endothelial cells selectively kill CD8+ T cells rather than regulatory T cells and establish tumor immune tolerance (119). Some cells educated in tumor microenvironments such as cancer-associated fibroblast (CAF), myeloid-derived suppressor cell (MDSC), microglia, and macrophages also express CD95L (120–122). Therefore, inhibition of the CD95-CD95L pathway in the tumor microenvironment may be a potential therapeutic strategy to improve the efficacy of immunotherapy.

## CD95 targeted therapy

Due to the typical pro-apoptotic function of CD95, targeting of the CD95 signaling pathway has been the focus of cancer therapy research. However, CD95 agonists have severe hepatotoxicity (123). To avoid the toxicity caused by its Fc segment, “Mega-Fas-Ligand”, a synthetic CD95 ligand, now known as APO010, was developed. The potential of APO010 to induce human glioma cell apoptosis has been observed *in vitro* (124, 125), but it is less effective than expected *in vivo* (126). A phase I trial (ClinicalTrials.gov Identifier: NCT00437736) is underway to determine the recommended dose of APO010.

As the understanding of CD95 tumorigenesis and immune cell killing functions has matured in recent years, inhibition rather than activation of CD95 signaling seems to be a better solution for GBM treatment. APG101 (also known asasunercept) is composed of the extracellular domain of CD95 and the Fc segment of the IG1 antibody, and acts as a CD95 inhibitor. Therefore, APG101 can specifically block CD95L on the tumor cell membrane and the vascular endothelial cell membrane, and the function of CD95L in promoting the invasion and apoptosis of activated T lymphocytes by glioma cells can be neutralized (127). We summarize the phase I/II clinical studies of APG101 in **Table 1**. Early trials in healthy subjects have demonstrated its safety (128). A phase I study showed that APG101 treatment significantly improved PFS with patients with GBM compared to standard radiotherapy and temozolomide alone, and hypomethylation of the CpG2 site within the CD95L promoter resulted in improved benefits (132).

**Table 1:** Phase I/II trials of APG101 and their results.

Subjects	Design	Safety and tolerability	Efficacy	Reference
Healthy men between the ages of 18 and 45	Volunteers were randomized to 7 drug dose-escalating groups (20 for asunercept, 14 for placebo).	60.0% patients who received Asunercept reported 19 AEs with an occurrence rate similar to the placebo group (57.1%). Only one possible treatment-related AE (headache) reported.	Unknown	(128)
Transfusion-dependent MDS patients with low or intermediate risk MDS	14 and 6 patients received 100 mg and 400 mg asunercept per week, respectively.	AEs occurred in 18 (90%) patients. 5 (25%) patients reported serious SAEs, 2 patients were considered experiencing treatment related AEs	Blood transfusion requirement of 20 patients decreased dramatically at 24 weeks after treatment.	(129)
Two patients with high-grade glioma	Patients received asunercept *via* intravenous infusion in different doses.	1 patient with GBM remained stable for 12 weeks. Relevant data for patient with anaplastic oligodendroglioma WHO grade III was unavailable.	Unknown	(128)
Patients with first or second progression of GBM	26 patients received reirradiation alone and 58 patients received reirradiation combined with asunercept.	No impairment of QoL was observed. Most patients tolerated reirradiation plus asunercept treatment well.	Treatment with asunercept plus reirradiation significantly prolonged TtD compared with reirradiation alone PFS-6 was 3.8% in the reirradiation group, while PFS-6 in the reirradiation plus asunercept group increased to 20.7%	(130, 131)
Newly-diagnosed glioblastoma	3 patients received low dose (200 mg/week) of asunercept combined with standard radiotherapy/temozolomide, while 7 patients received high dose (400 mg/week) of asunercept combined with standard radiotherapy/temozolomide.	68 AEs were reported in 10 people, mainly hair loss (60%) and constipation (60%). Only one possibly treatment related AE (Grade 1 gingival) was considered.	High dose of asunercept increased PFS-6 from 33.3% to 57.1%. In the high dose group, 4 of 7 patients had no disease progression after one year.	(132)

AEs, adverse events; SAEs, serious adverse events; QoL, quality of life; TtD, time to deterioration; PFS-6, 6-month progression-free survival.

## Discussion

CD95-mediated apoptosis and anti-apoptotic signaling within the tumor, contribute to the suppression of apoptotic pathways and the enhancement of survival pathways. Tumor cells also use CD95L expressed in the microenvironment to kill immune cells. Given the above understanding, inhibiting rather than activating the CD95 signals is a better strategy. Because CD95 and its downstream signaling molecules are involved in multiple pathways, direct targeting of effector molecules is also a good option, such as thermal therapy, which promotes the generation of the generation of cytochrome C to induce apoptosis in cancer cells (133). Another factor to consider is the complexity of the interaction between CD95 signaling and other pathways of cell death, such as caspase-8 activation to inhibit necrotizing apoptosis (134). Nonetheless, apoptosis and the cell cycle are closely related and their mutual influence should not be ignored.

Knockdown of CD95-CD95L signaling by si/shRNA induces tumor cell death, this phenomenon called Death-induced by CD95R/L elimination (DICE) has been reported in tumors (135). Putzbach et al. updated the DICE concept to Death-induced by survival gene elimination (DISE), confirming that cell death is not actually due to CD95 or CD95L knockdown. In fact, si/shRNA derived from CD95/CD95L silenced a set of surviving genes through a specific 6mer seed sequence (position 2-7 of the guide strand), resulting in the DISE (136). Furthermore, it has been confirmed that the expression of CD95L mRNA itself was toxic to cells through DISE (137). This could explain the inefficiency of exogenous CD95/CD95L or CD95 agonists, as they fail to trigger DISE. DISE relies on the involvement of RNA induced silencing complex (RISC) where miRNAs target mRNAs. Therefore nonspecific down-regulation of miRNA levels in cancer cells allows the release of RISCs occupied by them and then triggers DISE; whereas in normal tissues, most highly expressed miRNAs carry seed regions with low guanine content, making them less toxic to survival genes (138). Thus, DISE may induce cancer cell death. *In vivo* induction experiments with DISE have confirmed that normal tissues were not affected by toxicity, thus providing a partial basis for the safety of the strategy (139). As DISE toxicity is independent of CD95L or CD95 receptor expression, the combination of induced overexpression of CD95L and inhibition of CD95 signaling could represent a potential cancer treatment in the future.

## Author contributions

YZ was responsible for manuscript writing; TJ contributed to the organization of content; ZD and BW collected relevant data; BZ provided guidance on pathology; CS provided ideas, reviewed, and edited the draft. All authors contributed to the article and approved the submitted version.

## Funding

This work was supported by Zhejiang Science and Technology Plan Project Key R&D Plan (2021C03067).

## Acknowledgments

We wish to thank Qimi Cai from Zhejiang University for support and encouragement of this review. All figures in this review were created with BioRender.com.

## Conflict of interest

The authors declare that the research was conducted in the absence of any commercial or financial relationships that could be construed as a potential conflict of interest.

## Publisher’s note

All claims expressed in this article are solely those of the authors and do not necessarily represent those of their affiliated organizations, or those of the publisher, the editors and the reviewers. Any product that may be evaluated in this article, or claim that may be made by its manufacturer, is not guaranteed or endorsed by the publisher.

## Abbreviations

GBM, Glioblastoma; TNF, Tumor necrosis factor; NK, Natural killer; CRD, Cysteine-rich domains; FADD, Fas-associated protein with death domain; DED, Death effector domain; DISC, Death-inducing signaling complex; tBid, Truncated form of the BH3 interacting domain death agonist; MOMP, Mitochondrial outer membrane permeabilization; IκB, Inhibitor of NF-κB protein; IKK, IκB kinase; MMP, Matrix metalloproteinases; DcR, Decoy receptor; TRAIL, Tumor necrosis factor-related apoptosis-inducing ligand; Daxx, Death domain-associated protein; JNK, Jun NH2-terminal kinase; sCD95L, Soluble CD95L; MISC, Motion-induced signaling complex; mCD95L, Membrane-bound CD95L; IAP, Inhibitors of apoptosis proteins; DISE, Death-induced by survival gene elimination.
